# Seronegative Autoimmune Encephalitis With Neuropsychiatric Presentation: A Case Report

**DOI:** 10.7759/cureus.104133

**Published:** 2026-02-23

**Authors:** Christian David Galindo, Lesmer Galindo Ruiz, Victor Hugo Agudelo, Natalia Mejia, Uriel Castro Diaz

**Affiliations:** 1 Psychiatry, Remington University Corporation, Medellín, COL; 2 Psychiatry and Behavioral Sciences, Mental Antioquia Hospital, Medellín, COL; 3 Psychiatry and Behavioral Sciences, Remington University Corporation, Medellín, COL; 4 Hospital Medicine, Hospital Internacional de Colombia, Santander, COL

**Keywords:** autoimmune encephalitis negative serotype, neuropsychiatric manifestations, paraneoplastic neurologic syndrome, psychiatry and neuroscience, seronegative autoimmune encephalitis

## Abstract

Seronegative autoimmune encephalitis (AE) is an immune-mediated inflammatory disorder of the central nervous system that presents with a broad spectrum of neuropsychiatric manifestations, including acute behavioral changes, cognitive dysfunction, catatonia, and altered levels of consciousness. The absence of identifiable neuronal autoantibodies in serum or cerebrospinal fluid contributes to diagnostic uncertainty, delayed treatment initiation, and increased morbidity. Given the potential reversibility of this condition, early recognition and prompt initiation of immunotherapy are critical to optimizing clinical outcomes. We report the case of a 68-year-old woman with probable seronegative AE who presented with subacute neuropsychiatric deterioration characterized by prominent psychiatric symptoms and catatonia. An extensive diagnostic evaluation, including cerebrospinal fluid analysis, comprehensive autoimmune antibody testing, and exclusion of infectious, metabolic, and structural etiologies, failed to identify an alternative diagnosis. Functional neuroimaging with 18F-fluorodeoxyglucose positron emission tomography revealed cerebral metabolic abnormalities supportive of an inflammatory encephalitic process. The patient demonstrated marked clinical improvement following early initiation of high-dose intravenous corticosteroid therapy. This case underscores the diagnostic complexity of seronegative AE when neuropsychiatric manifestations predominate and overlap with primary psychiatric disorders. It highlights the importance of maintaining a high index of clinical suspicion despite negative serological findings and supports the use of clinical diagnostic criteria and adjunctive functional neuroimaging to guide early immunotherapeutic intervention in suspected seronegative AE.

## Introduction

Autoimmune encephalitis (AE) comprises a group of non-infectious, immune-mediated inflammatory disorders of the central nervous system characterized by rapidly progressive encephalopathy secondary to widespread autoimmune-driven neuroinflammation [1]. AE represents a significant diagnostic challenge, as its clinical presentation, laboratory findings, and neuroimaging features frequently overlap with those of infectious encephalitis, metabolic disturbances, and primary psychiatric disorders [2]. Characteristic clinical presentations encompass a broad spectrum of acute to subacute neuropsychiatric and neurological manifestations, including altered mental status, cognitive impairment, seizures, focal neurological deficits, psychosis, and coma. Although historically regarded as a rare condition, emerging epidemiological evidence indicates that AE occurs with a frequency comparable to that of infectious encephalitis, with an estimated incidence of approximately 13.7 per 100,000 individuals [3].

The pathogenesis of AE arises from an aberrant immune response directed against self-antigens expressed within the central nervous system. Based on immunopathological mechanisms and clinical features, AE encompasses a heterogeneous group of disorders that can be conceptually classified according to immunopathological mechanisms and clinical features, including antibody-mediated forms, cell-mediated forms, and seronegative cases [4]. These syndromes may result from paraneoplastic immune responses involving antibodies against intracellular neuronal antigens; autoantibodies targeting extracellular receptors, ion channels, or synaptic proteins; or immune-mediated mechanisms driven by autoantibodies that have not yet been clearly identified [5].

Seronegative AE represents a particularly challenging subtype of AE and is defined by the absence of identifiable pathogenic autoantibodies in cerebrospinal fluid or serum despite a clinical phenotype consistent with AE. The lack of detectable antibodies is currently hypothesized to reflect the presence of unidentified or yet to be discovered autoantibodies, as well as heterogeneous immune mechanisms not captured by available diagnostic assays [6]. Consequently, the diagnosis of seronegative AE relies predominantly on clinical assessment and supportive paraclinical findings, which are often limited by reduced sensitivity and considerable heterogeneity [7]. This diagnostic uncertainty frequently leads to delayed recognition and initiation of immunotherapy, particularly in cases in which neuropsychiatric manifestations predominate and obscure the underlying neurological etiology [8].

This case report is presented to illustrate the diagnostic challenges associated with seronegative AE presenting with prominent neuropsychiatric manifestations and to emphasize the importance of maintaining a high index of clinical suspicion to facilitate timely diagnosis and appropriate immunomodulatory management.

## Case presentation

A 68-year-old female with a medical history significant for type II diabetes mellitus, arterial hypertension, dyslipidemia, hypothyroidism, sinus bradycardia with permanent pacemaker implantation, and structural aortic valve disease status post-bioprosthetic aortic valve replacement presented to the emergency department with progressive altered mental status and prominent neuropsychiatric deterioration. According to family members, the patient had been in her usual state of health and fully independent in activities of daily living until several weeks before presentation, when she developed insidious behavioral and cognitive changes. These initial symptoms included progressive disorientation, impaired attention, psychomotor slowing, reduced spontaneous speech, and social withdrawal. Over subsequent days, her clinical course evolved with the emergence of marked psychiatric manifestations, including paranoid ideation, auditory hallucinations, emotional blunting, and episodes of mutism. The condition further progressed with fluctuating levels of consciousness, rigidity, immobility, negativism, and reduced oral intake, raising concern for a catatonic syndrome. Due to progressive functional decline and worsening mental status, the patient was brought to the hospital for further evaluation.

Upon arrival at the emergency department, the patient was afebrile and hemodynamically stable. Mental status examination revealed fluctuating alertness, partial orientation to person, disorientation to time and place, marked attentional deficits, bradypsychia, impaired recent memory, hypophonic and sparse speech, and pronounced psychomotor retardation. Affect was markedly flattened with poor reactivity. Neurological examination demonstrated generalized rigidity without focal motor deficits or meningeal signs. Given the acute-to-subacute neuropsychiatric presentation, the initial differential diagnosis included delirium, a primary psychiatric disorder with psychotic features, catatonia, and an underlying neurological or inflammatory encephalopathic process.

Initial laboratory evaluation, including complete blood count, metabolic panel, liver and renal function tests, inflammatory markers, and infectious screening, revealed no significant abnormalities. Cerebrospinal fluid analysis demonstrated marked hyperproteinorrachia with normal glucose levels and absence of pleocytosis. Cerebrospinal fluid cytology and cell block analysis were negative for malignant or atypical cells. Extensive infectious cerebrospinal fluid studies were negative for herpes simplex virus, varicella-zoster virus, Epstein-Barr virus, cytomegalovirus, and other common neurotropic pathogens. Comprehensive serum and cerebrospinal fluid autoimmune and paraneoplastic antibody panels were negative, including antibodies against N-methyl-D-aspartate receptor, α-amino-3-hydroxy-5-methyl-4-isoxazolepropionic acid receptor, gamma-aminobutyric acid type B receptor, leucine-rich glioma-inactivated 1, contactin-associated protein-like 2, glutamic acid decarboxylase 65-kDa isoform, Hu, Yo, Ri, Ma2, and CV2. Evaluation for prion disease, including cerebrospinal fluid 14-3-3 protein and RT-QuIC assay, was negative. Relevant laboratory and cerebrospinal fluid findings are summarized in Table 1.

**Table 1:** Relevant laboratory and cerebrospinal fluid findings. CSF: cerebrospinal fluid; WBC: white blood cell count; HSV: herpes simplex virus; VZV: varicella-zoster virus; EBV: Epstein–Barr virus; CMV: cytomegalovirus; CRP: C-reactive protein; ESR: erythrocyte sedimentation rate; TSH: thyroid-stimulating hormone; PCR: polymerase chain reaction; NMDAR: N-methyl-D-aspartate receptor; AMPAR: α-amino-3-hydroxy-5-methyl-4-isoxazolepropionic acid receptor; GABA-B: gamma-aminobutyric acid type B receptor; LGI1: leucine-rich glioma-inactivated 1; CASPR2: contactin-associated protein-like 2; DPPX: dipeptidyl-peptidase-like protein 6; GlyR: glycine receptor; RT-QuIC: real-time quaking-induced conversion; CRMP5: collapsin response mediator protein 5

Category	Test	Patient’s result	Reference range/Interpretation
Peripheral blood	White blood cell count (×10⁹/L)	7.8	4.5–11.0
	Serum glucose (mg/dL)	110	70–140
	Thyroid-stimulating hormone (TSH, mIU/L)	4.1	0.5–5.0
	Free T4 (ng/dL)	1.1	0.8–1.8
	C-reactive protein (mg/L)	1.2	<5.0
	Erythrocyte sedimentation rate (mm/h)	12	<20
Cerebrospinal fluid analysis	Opening pressure (mmHg)	12	10–20
	White blood cells (cells/mm³)	0	0–5
	Differential	No pleocytosis	Lymphocytic predominance in inflammatory states
	Protein (mg/dL)	180	15–40
	Glucose (mg/dL)	65	45–80
CSF infectious studies	HSV-1/2 PCR	Negative	Negative
	VZV PCR	Negative	Negative
	EBV PCR	Negative	Negative
	CMV PCR	Negative	Negative
CSF autoimmune studies	Neuronal surface antibodies (NMDAR, AMPAR, GABA-B, LGI1, CASPR2)	Negative	Negative
	Other neuronal antibodies (DPPX, GlyR)	Negative	Negative
Serum autoimmune studies	Neuronal surface antibodies (NMDAR, AMPAR, GABA-B, LGI1, CASPR2)	Negative	Negative
	Onconeural antibodies (Hu, Yo, Ri, Ma2, CV2/CRMP5)	Negative	Negative
Neoplastic evaluation	CSF cytology and cell block	Negative	No malignant or atypical cells detected
Prion disease evaluation	CSF 14-3-3 protein	Negative	Negative
	RT-QuIC assay	Negative	Negative

**Figure 1:**
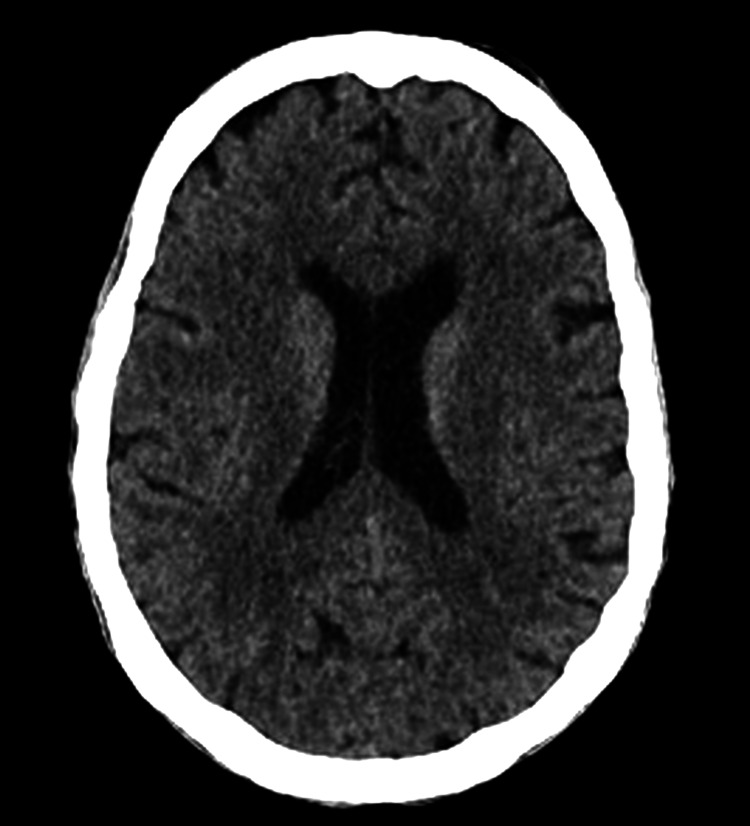
Non-contrast CT of the brain. Non-contrast axial CT of the brain demonstrating preserved gray–white matter differentiation and symmetric ventricular system, with no evidence of acute intracranial hemorrhage, mass effect, or ischemic lesions. These findings highlight the limited sensitivity of structural neuroimaging in the early stages of autoimmune encephalitis, despite significant neuropsychiatric manifestations.

Initial neuroimaging with non-contrast CT of the brain revealed no evidence of acute ischemia, hemorrhage, or mass lesions, failing to account for the severity of the patient’s neuropsychiatric presentation (Figure 1).

**Figure 2:**
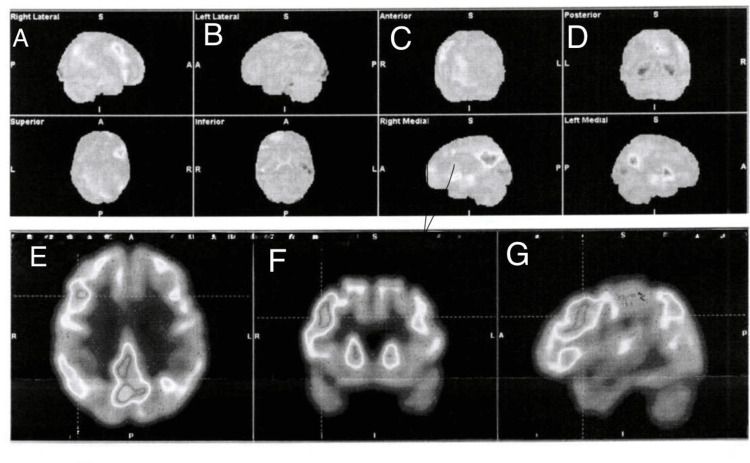
Brain fluorodeoxyglucose positron emission tomography (FDG-PET). Brain FDG-PET demonstrates a pattern of regional metabolic abnormalities consistent with an active inflammatory encephalitic process. Panels A–G correspond to three-dimensional cortical surface projections, including right lateral (A), left lateral (B), anterior (C), posterior (D), superior (E), inferior (F), and right medial (G) views. The study reveals marked hypermetabolism in the right frontal lobe, predominantly involving the superior frontal gyrus and frontobasal regions, as well as bilateral hypermetabolism of the posterior cingulate cortex, findings suggestive of focal cortical hyperexcitability and active neuroinflammation. In contrast, reduced metabolic activity is observed in the bilateral temporal and occipital cortices, with left-sided predominance, along with focal hypometabolism in the left parietal region, a pattern consistent with cortical dysfunction secondary to inflammatory involvement. Metabolic activity is preserved in the basal ganglia and thalami, with mild asymmetry in cerebellar metabolism. The combination of frontal–cingulate hypermetabolism and posterior cortical hypometabolism, in the absence of corresponding structural abnormalities on conventional neuroimaging, is characteristic of autoimmune encephalitis and supports an active inflammatory encephalitic process.

Subsequent MRI of the brain similarly demonstrated no acute or subacute infarction and no focal inflammatory lesions. Given the persistence of symptoms and high clinical suspicion for an underlying neuroinflammatory process, functional neuroimaging was pursued. Fluorodeoxyglucose positron emission tomography revealed marked hypermetabolism involving frontal cortical regions and the posterior cingulate cortex, a metabolic pattern consistent with active AE and strongly supportive of an inflammatory encephalopathic process (Figure 2).

A comprehensive oncological evaluation, including contrast-enhanced CT of the chest, abdomen, and pelvis, revealed no evidence of underlying malignancy. An incidental uterine leiomyoma with benign radiological characteristics was identified and interpreted as a non-contributory finding, without clinical or radiological features suggestive of paraneoplastic involvement; consequently, neither biopsy nor surgical intervention was considered.

Despite the absence of detectable neuronal autoantibodies, the overall clinical picture, characterized by subacute neuropsychiatric deterioration, catatonia-like features, encephalopathic electroencephalographic abnormalities, an inflammatory cerebrospinal fluid profile, and characteristic metabolic alterations on positron emission tomography, was highly suggestive of seronegative AE. The patient was subsequently initiated on immunomodulatory therapy. Plasma exchange was administered every other day, completing a total of six sessions. Initial clinical improvement became apparent after the fourth plasma exchange session, with gradual restoration of attention, spontaneous speech, and psychomotor activity. Upon completion of plasma exchange, intravenous methylprednisolone at a dose of 500 mg daily was administered for five consecutive days. In light of the favorable clinical response, maintenance immunosuppression with oral prednisone at a dose of 60 mg daily was subsequently initiated.

Cognitive and neuropsychiatric follow-up was systematically conducted using serial Montreal Cognitive Assessment (MoCA) testing, complemented by repeated comprehensive mental status and neurological examinations performed at three-day intervals. Before initiation of immunomodulatory therapy, the patient demonstrated significant cognitive impairment, with an initial MoCA score of 12/30. Following three days of treatment, the score improved to 15/30, and after completion of plasma exchange, further improvement was observed with a score of 18/30. At the conclusion of corticosteroid therapy, the MoCA score reached 25/30, consistent with substantial cognitive recovery. At follow-up, corresponding to a total hospitalization period of 25 days from admission until complete resolution of neuropsychiatric symptoms, the patient had returned to her baseline mental status and functional capacity.

## Discussion

This case illustrates a clinically relevant presentation of probable seronegative AE with predominant neuropsychiatric involvement, highlighting the diagnostic complexity inherent to antibody-negative cases and underscoring the central role of clinical reasoning in guiding timely therapeutic decisions [9]. Neuropsychiatric manifestations such as acute behavioral changes, catatonia, and alterations in the level of consciousness are increasingly recognized as core features of AE and may precede or obscure overt neurological deficits [10].

Despite extensive cerebrospinal fluid and serum testing, no neuronal surface or synaptic autoantibodies were identified. However, seronegativity does not exclude AE. Available epidemiological evidence suggests that a substantial proportion of encephalitis cases remain etiologically undefined, and that a meaningful subset of AE cases are antibody-negative, likely reflecting immune-mediated mechanisms or pathogenic antibodies not yet identified by current diagnostic assays [11]. Consequently, exclusive reliance on antibody testing may contribute to diagnostic delay and negatively impact clinical outcomes.

Contemporary diagnostic frameworks support the diagnosis of probable AE in seronegative patients when a compatible clinical syndrome is present and alternative infectious, metabolic, structural, and toxic causes have been reasonably excluded. In this case, the subacute onset of prominent psychiatric symptoms, the presence of catatonia, supportive ancillary findings, and a clear clinical response to immunotherapy fulfilled these criteria, supporting early initiation of immunosuppressive treatment despite negative antibody results.

Catatonia represents a clinically significant neuropsychiatric phenotype within the AE spectrum and constitutes an important diagnostic red flag. It has been described across multiple AE subtypes and is frequently misattributed to primary psychiatric disorders, particularly when overt neurological signs are absent [12]. The coexistence of catatonia with fluctuating mental status, limited response to antipsychotic therapy, or autonomic features should heighten suspicion for an underlying autoimmune process. In this patient, catatonic symptoms showed a favorable response to lorazepam, consistent with established symptomatic management, while sustained clinical improvement following immunomodulatory therapy supports an immune-mediated pathophysiological mechanism [13].

Immunotherapy remains the cornerstone of treatment for AE, regardless of serological status. First-line therapeutic strategies include high-dose intravenous corticosteroids, intravenous immunoglobulin, plasmapheresis, or combination regimens [14]. Although treatment recommendations for seronegative AE are largely based on observational studies and expert consensus, early initiation of immunotherapy has consistently been associated with improved neurological and functional outcomes [15]. In the present case, prompt administration of high-dose intravenous corticosteroids resulted in marked clinical improvement, reinforcing the importance of early intervention once AE is suspected.

In patients who do not demonstrate adequate clinical response to first-line therapy, escalation to second-line immunosuppressive agents such as rituximab or cyclophosphamide is recommended, with rituximab generally preferred due to its more favorable safety and tolerability profile [16]. Importantly, delays in immunotherapy initiation have been associated with worse cognitive and functional recovery, further emphasizing the need for timely recognition and decisive management.

Neuroimaging findings in AE are frequently normal or nonspecific on conventional MRI, particularly during early stages of disease. Growing evidence supports the diagnostic utility of FDG-PET/CT as a functional imaging modality capable of detecting cerebral metabolic abnormalities when MRI findings are inconclusive [17]. Reported metabolic patterns, including cortical hypometabolism or regional hypermetabolism, have been shown to correlate with neuropsychiatric manifestations such as catatonia and behavioral disturbances, suggesting that FDG-PET may serve as a valuable adjunct in the diagnostic evaluation of suspected seronegative AE [18].

From a clinical perspective, this case contributes to the existing literature by reinforcing the importance of recognizing catatonia as a potential manifestation of AE in the absence of detectable autoantibodies, and by illustrating the complementary diagnostic value of functional neuroimaging alongside clinical criteria. It further underscores that early immunotherapy, guided by clinical suspicion rather than antibody status alone, may lead to favorable outcomes and prevent prolonged morbidity.

## Conclusions

Seronegative AE represents a diagnostic challenge due to the absence of identifiable neuronal autoantibodies despite a clinically compatible neuropsychiatric presentation. In this case, the subacute onset of prominent psychiatric symptoms with catatonia, supportive ancillary findings, and a favorable response to immunotherapy supported the diagnosis of probable AE despite negative serological testing. Negative antibody results should not delay diagnosis or treatment when clinical suspicion is high, and early initiation of first-line immunotherapy is essential to achieve clinical improvement. In patients with insufficient response, escalation to second-line immunosuppressive therapy should be considered to optimize outcomes. Additionally, FDG-PET may serve as a valuable adjunct in the diagnostic evaluation of suspected seronegative AE, particularly when conventional neuroimaging is inconclusive.
